# Recent Insights into the Role of B Cells in Chronic Hepatitis B and C Infections

**DOI:** 10.3390/pathogens12060815

**Published:** 2023-06-08

**Authors:** Zgjim Osmani, Andre Boonstra

**Affiliations:** Department of Gastroenterology and Hepatology, Erasmus University Medical Center, 3015 GD Rotterdam, The Netherlands

**Keywords:** B cells, chronic viral hepatitis, hepatitis B, hepatitis C

## Abstract

Chronic viral hepatitis infections, caused by the hepatitis B or C virus, are a major global health problem causing an estimated one million deaths each year. Immunological studies have classically focused on T cells, while B cells have largely been neglected. Emerging evidence, however, highlights a role for B cells in the immunopathogenesis of chronic hepatitis B and C infections. B cell responses appear to be altered across different clinical phases of chronic HBV infection and across stages of disease in chronic HCV infection. These B cell responses show signs of a more activated state with a simultaneous enrichment of phenotypically exhausted atypical memory B cells. Despite the fact that studies show an activating B cell signature in chronic viral hepatitis infection, antibody responses to HBsAg remain impaired in chronic HBV infection, and glycoprotein E2-specific neutralizing antibody responses remain delayed in the acute phase of HCV infection. At the same time, studies have reported that a subset of HBV- and HCV-specific B cells exhibit an exhausted phenotype. This may, at least in part, explain why antibody responses in chronic HBV and HCV patients are suboptimal. Here, we summarize recent findings and discuss upcoming research questions while looking forward to how new single-cell technologies could provide novel insights into the role of B cells in chronic viral hepatitis infections.

## 1. Introduction

Chronic infections with hepatitis viruses remain a major global health problem, in particular, infections with the hepatitis B virus (HBV) and hepatitis C virus (HCV), which may cause liver cirrhosis, end-stage liver disease, and hepatocellular carcinoma (HCC) [1]. Worldwide, as many as an estimated 296 million people have a chronic HBV infection and 58 million have a chronic HCV infection, and about one million people die each year from the consequences of these infections [2]. HBV and HCV belong to the Hepadnaviridae and Flaviviridae families, respectively. HBV is a small, enveloped, partially double-stranded DNA virus, which consists of a nucleocapsid core containing the viral genome and a surrounding envelope that is made up of host-cell-derived lipids and viral proteins. The viral genome of HBV is approximately 3.2 kilobases in length and encodes the core antigen (HBcAg), the surface antigen (HBsAg), the hepatitis B e antigen (HBeAg), the polymerase, and the X protein [3,4]. HCV, on the other hand, is a small, enveloped, single-stranded highly mutable RNA virus with a genome of approximately 9.6 kilobases in length that encodes a single polyprotein which is cleaved by host and viral proteases to generate ten mature proteins, including structural proteins (core, E1-, and E2 envelope glycoproteins) and non-structural proteins (p7, NS2, NS3, NS4A, NS4B, NS5A, and NS5B). Structural proteins are responsible for the formation of the viral particle, while non-structural proteins are involved in viral replication and assembly [4,5]. Both the HBV envelope (HBsAg) and the HCV envelope glycoproteins (E1/E2) are the major targets of the host immune response. Humoral immune responses to HBsAg lead to a successful prophylactic HBV vaccination response or recovery from acute HBV infection [3]. Vaccination programs have been implemented globally for HBV, which have resulted in a significant decline in HBV transmission and HBV-related complications worldwide [6,7]. HBV exclusively infects hepatocytes through the interaction of HBsAg with sodium taurocholate co-transporting polypeptide (NTCP) and its co-receptor epidermal growth factor receptor (EGFR). It is also noteworthy that recent evidence suggests that HBV associates with lipoproteins to access the perisinusoidal space or to follow the physiological cholesterol transport pathway to hepatocytes via macrophages [8]. After membrane fusion, the HBV genome is transported to the nucleus, where it is converted into a highly stable covalently closed circular DNA (cccDNA) molecule that serves as a template for viral gene transcription and replication [9,10]. The replication of HBV occurs through an unusual reverse transcription mechanism, where the viral polymerase synthesizes a negative-sense DNA strand from the pgRNA template, followed by the synthesis of a positive-sense DNA strand that serves as the template for the production of viral RNA transcripts. The viral RNA transcripts are then translated into viral proteins, which are assembled into viral particles and released from the infected hepatocytes [10,11]. It is generally accepted that HCV also exclusively infects hepatocytes, although some incidental studies have suggested that immune cells, including B cells, can also be infected leading to upregulated B cell receptor signaling, which may cause B cell dysregulation and abnormalities [12,13,14]. However, it needs to be stressed that more studies are needed to prove the non-hepatocyte route. HCV enters the host cell through the interaction of its envelope glycoproteins with various host cell receptors, including claudin-1, upon which the positive-sense RNA viral genome is released into the cytoplasm that serves as the template for viral gene translation and replication [15,16]. HCV RNA is then translated into a single polyprotein, which is cleaved into individual proteins by proteases [16]. HBV and HCV have distinct mechanisms of virus assembly and release. HBV virions are assembled in the endoplasmic reticulum (ER) and Golgi complex of infected hepatocytes. The HBcAg forms the viral capsid that encloses the viral genome and other viral proteins. The viral envelope is formed by the HBsAg, which is synthesized as a glycosylated precursor protein and transported to the ER, where it is cleaved and assembled into virions. Mature HBV virions are released from the infected hepatocytes by budding through the ER and Golgi membranes [3,11]. HCV virion assembly occurs on specialized intracellular membrane structures known as the membranous web, which are formed by the rearrangement of host cell membranes induced by viral proteins. The HCV core protein is the major component of the viral nucleocapsid, which surrounds the viral RNA genome. The envelope glycoproteins E1 and E2 are synthesized as glycosylated precursor proteins and transported to the ER, where they are cleaved and assembled into virions. The mature HCV virions are then released from the infected cells by a non-lytic mechanism that involves the formation of secretory vesicles transporting the virions to the cell surface, where they are released [5,16].

## 2. Natural History and Immunology of Viral Hepatitis Infections

The liver is a vital organ with diverse metabolic and clearance functions, but it also contains unique immune regulatory properties; hepatocytes in the liver are the primary target of HBV and HCV. Both are non-cytopathic viruses that make use of these tolerogenic properties of the liver [4]. Although both viruses can cause acute and chronic infections, there are significant differences in the course and stages of infection, clinical phases, and immunological response. During the acute phase of HBV infection, patients may experience symptoms, and in more than 95% of immunocompetent adults, the infection resolves spontaneously and patients develop protective immunity [17]. However, in some cases, acute infection can progress to lifelong chronic infection. The risk of progression to chronic infection is highest in infants and young children; up to 95% of perinatally infected infants develop chronic infections [10,17]. Unlike HBV, most patients in the acute phase of HCV infection are asymptomatic and develop a chronic infection; only 18–34% of patients spontaneously clear an acute HCV infection [18].

**Figure 1:**
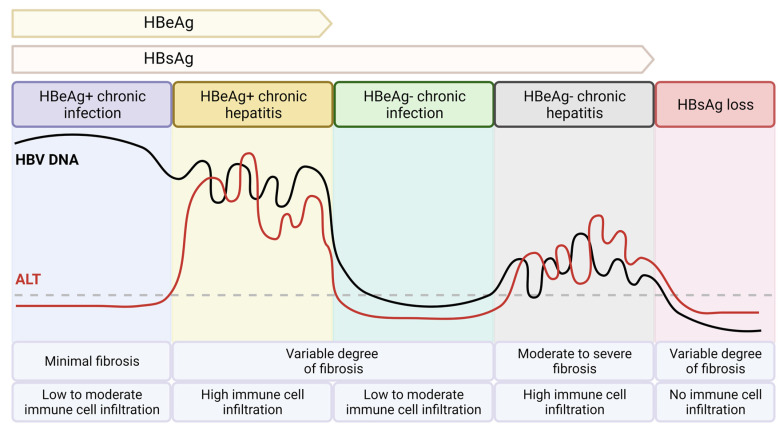
Clinical phases of chronic HBV infection.

**Figure 2:**
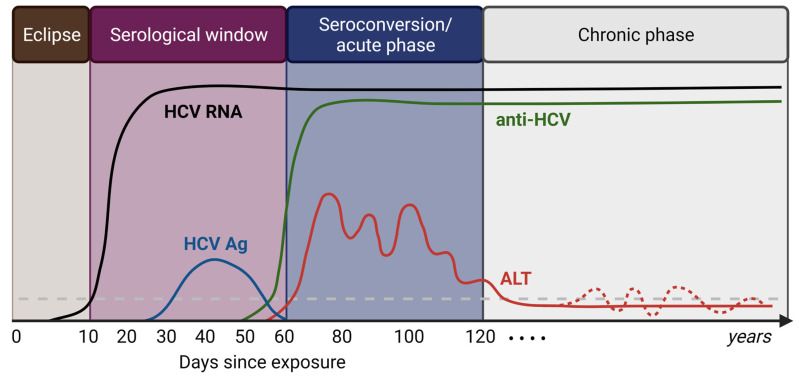
Natural history of HCV infection.

The course of a chronic HBV and HCV infection also differs. Chronic HBV patients experience episodes with fluctuating serum levels of viral DNA and alanine transaminase (ALT) liver enzyme levels (Figure 1), while a chronic HCV infection is generally quite stable when assessing these parameters (Figure 2). Patients with a chronic HBV infection are classified into five clinical phases: HBeAg-positive chronic infection, HBeAg-positive chronic hepatitis, HBeAg-negative chronic infection, HBeAg-negative chronic hepatitis, and HBsAg loss phase [19]. In the HBeAg-positive chronic infection phase, patients have high levels of HBV DNA, normal ALT levels, and are HBeAg-positive. In the HBeAg-positive chronic hepatitis phase, patients are still HBeAg-positive and have relatively high immune cell infiltration in the liver with concomitant elevated serum HBV DNA and liver enzyme levels reflecting liver damage. In the HBeAg-negative chronic infection phase, patients become HBeAg-negative and have a stable chronic infection with low levels of HBV DNA and normal liver enzyme levels. In the HBeAg-negative chronic hepatitis phase, patients remain HBeAg-negative and experience a flare in liver disease, showing again relatively high immune cell infiltration with elevated serum HBV DNA and liver enzyme levels. The HBsAg loss phase is characterized by the absence of serum HBeAg and HBsAg, undetectable HBV DNA levels, and normal ALT levels. HBsAg loss is associated with a lower risk of liver cirrhosis, decompensation, and HCC, and is therefore regarded as the optimal treatment endpoint for patients with chronic HBV infections [19]. Of note, the different clinical phases of chronic HBV infection do not always occur sequentially. In contrast, chronic HCV infections are characterized by a single chronic phase with relatively stable levels of virus and liver enzyme activity and persistent hepatic inflammation, leading to the development of liver cirrhosis in approximately 10–20% of patients [18]. The clinical phases of chronic HBV infection reflect the dynamic interplay between the virus and the host immune system. At first encounter, immune responses play a critical role in the clearance of an acute HBV infection, characterized by the production of antibodies against HBsAg (anti-HBs); however, immune responses to HBV and their role in the pathogenesis of hepatitis B are incompletely understood [10]. During a chronic HBV infection, immune responses are often weak or ineffective, which allows viral replication and persistence in the liver [20]. From an immunological point of view, infections with HCV demonstrate a more pronounced induction of interferons and interferon-stimulated genes compared to HBV; however, virus-specific immune responses are functionally impaired in both chronic HBV and HCV infection [21].

## 3. Current Treatments and the Need for Novel Treatment Strategies

Interferon-alpha was the first drug used to treat HCV infection, but this was only effective in about half of the patients, depending on the HCV genotype [22]. The introduction of direct-acting antiviral agents (DAAs) has revolutionized treatment for chronic HCV infections since viral eradication is observed in almost all patients with negligible side effects [22]. For chronic HBV, treatment consists of interferon-alpha or antiviral therapy with nucleos(t)ide analogues (NUC), but this does not lead to the complete eradication of HBV [19]. The reason for this is that HBV persists as a mini-chromosome in the nucleus of infected hepatocytes and remains even during long-term antiviral therapy, which often necessitates lifelong treatment, but not always [23]. The prevention of infection through vaccination is therefore essential to further limit the spread of HBV [1,6]. In 2016, the World Health Organization published an ambitious plan that aims to eliminate viral hepatitis as a public health threat worldwide by 2030 [2]. Despite the availability of an effective vaccine for HBV and effective antiviral therapies for both HBV and HCV, access to antiviral medication is still limited, and many patients with chronic viral hepatitis infections still develop liver fibrosis, cirrhosis, and HCC [24]. In addition, the majority of people with chronic HBV infections have not been tested or diagnosed, and a substantial proportion of people carry occult HBV infection without knowing their infection status or treatment indication [25]. Although we are on the path towards the elimination of HBV and HCV, the lack of an effective preventive or therapeutic vaccine hampers worldwide eradication, and novel treatment strategies aimed at control or elimination are clearly needed [22,26,27,28]. Preventive or therapeutic vaccination exploiting the antigen presentation capacity of antigen-presenting cells to prime or boost immune responses to HBV or HCV is considered promising. However, the success of these approaches has been limited for a number of reasons, including the use of adjuvants, the delivery, the limited availability of animal models, the existence of multiple HCV genotypes, and the complex nature of the immunological response to HBV and HCV [29,30]. For an optimal effect of therapeutic HBV vaccination, it is likely essential to take a stepwise approach by first reducing the viral load with NUC therapy and/or siRNA-based therapeutic modalities [29,31]. To maximize vaccine efficacy for highly mutable RNA viruses such as HCV, it is thought that vaccine strategies should focus on targeting neutralizing epitopes of multiple HCV genotypes, generating cross-genotype B and T cell responses associated with the induction of broadly neutralizing antibodies [32,33]. So far, immunological studies have been critical in advancing our understanding of the natural history of chronic viral hepatitis infections and remain important for the development of novel therapeutic approaches [34,35,36]. Immunological studies of chronic viral hepatitis infections classically focus on T lymphocytes, while B cells have largely been neglected and understudied. However, emerging evidence has highlighted a role for B cells in the immunopathogenesis of chronic viral hepatitis infections. For HBV, multiple studies have shown that B cell depletion therapy with the anti-CD20 monoclonal antibody rituximab increases the risk of HBV reactivation [37,38,39,40,41,42], thereby suggesting that B cells may contribute to the continuous control of HBV. For HCV, the association of chronic infection with clonal B cell proliferation, immune-mediated abnormalities such as mixed cryoglobulinemia, and the development of B cell lymphoproliferative malignancies altogether suggest an important role for B cells in the pathogenesis of HCV infection [43]. Recent advances in the field have provided more detailed information on the role B cells play in chronic viral hepatitis infections. Here, we summarize recent findings and discuss upcoming research questions while looking forward to how new single-cell technologies could provide novel insights into the role of B cells in chronic viral hepatitis infections.

## 4. Gene Expression Studies Pointing towards a Role for B Cells in the Immunopathogenesis of Chronic Viral Hepatitis

The different clinical phases of a chronic HBV infection are instructive for clinicians to decide whether or not to start antiviral treatment since, generally, this is only provided in those phases with elevated serum ALT levels. However, the mechanisms underlying fluctuating viral replication and ALT levels have not been well-characterized. Using a microarray analysis of whole blood, we demonstrated that clinical phases of chronic HBV are characterized by distinct gene signatures in blood, with innate interferon genes and B cell response signatures being highly active during the HBeAg-positive chronic hepatitis phase [44]. It is important to note that there was no significant difference in total B cell frequencies in the blood of patients across different clinical phases of chronic HBV [44]. In chronic viral hepatitis infections, immune signatures in the liver may differ significantly from those observed in peripheral blood. B cell signatures were found to correlate with active clinical phases of chronic HBV not only in blood but also in the liver, as revealed by RNA-seq of liver biopsies [45,46,47]. Fluctuations in viral load and immune-mediated liver damage coincided with fluctuations in the intrahepatic transcriptome of patients with chronic HBV infections, pointing towards a more pronounced B cell transcriptome profile correlating with a higher inflammation status [45]. In HCV, RNA-seq B cell gene signatures in the blood of chronic HCV patients were found to be significantly reduced during the early acute stages of infection, most likely due to a reduction in the relative frequency of circulating B cells [48]. Previous studies performing RNA-seq on the liver biopsies of chronic HCV patients showed that a subset of patients have high gene expression levels of interferon-stimulated genes [49]. However, upon further investigation of gene expression changes in liver induced by HCV infection, gene signatures associated with B cell activation were shown to be significantly induced by HCV compared to non-infected controls [49]. To further unravel the role of B cells in the different phases of chronic HBV, our group performed RNA-seq on the purified B cells obtained from blood and liver showing unique differences between intrahepatic and peripheral B cells, pointing towards signs of a more activated state in the liver [50]. Moreover, a comparison of B cell transcriptomes in the blood from chronic HBV patients with different clinical phases versus healthy controls pointed towards a higher activation state of B cells in the HBeAg-positive chronic hepatitis phase, characterized by a higher gene expression of CD69 and CD83 [50]. In HCV, the RNA-seq of B cells in the peripheral blood of chronically infected patients showed significant enrichment of gene sets corresponding to type I interferon signaling and viral infection compared to healthy controls [51]. These gene expression studies in blood and liver collectively point towards a role for B cells in chronic HBV and HCV infection; however, the functions of B cells during chronic infection are not well understood.

## 5. Activating B Cell Signatures in Chronic Viral Hepatitis

**Figure 3:**
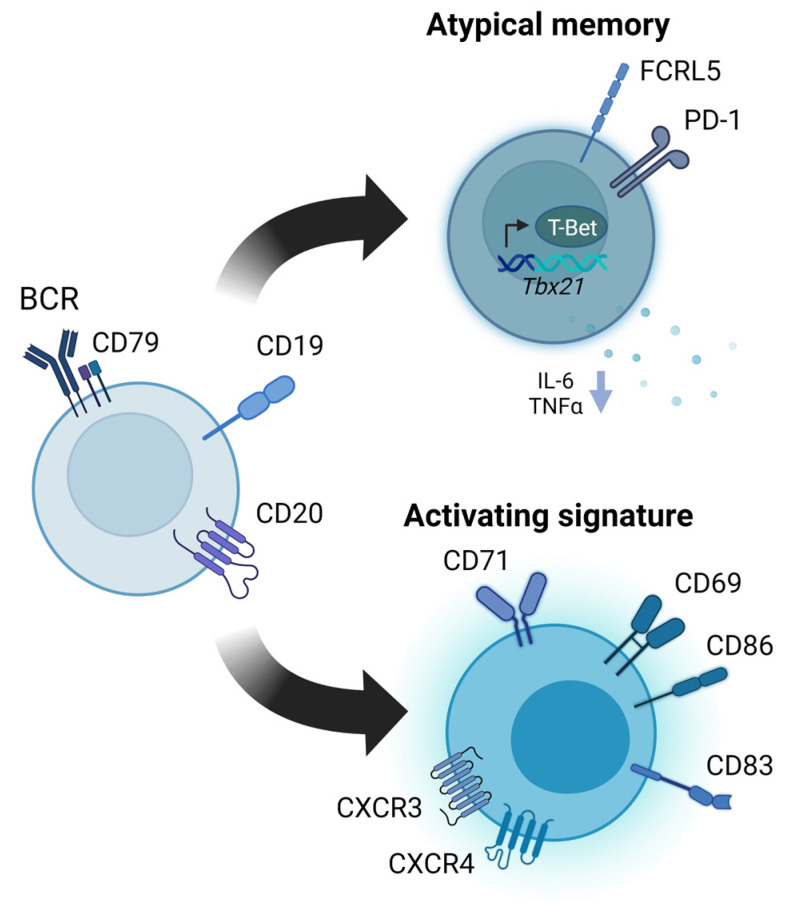
B cells in chronic viral hepatitis infections show signs of a more activated state with a simultaneous enrichment of phenotypically exhausted atypical memory B cells.

Evidence indicates that B cells are more activated during chronic infection with HBV and HCV relative to healthy controls [50,52,53,54,55,56,57,58,59]. The hyperactivated state is often characterized by a higher expression of activation markers CD69, CD71, CD83, and CD86 and chemokine receptors CXCR3 or CXCR4 (Figure 3). Elevated CD69 and CD71 protein levels on peripheral blood B cells have been described for both patients with chronic HBV and HCV infections [52,54,55,56,59], whereas the gene expression of CD83 was only highly expressed in HBV [50,53] and CD86 protein expression only in HCV-infected patients [54,55,56,59]. As mentioned earlier, we showed that B cells in the blood and liver of chronic HBV patients displayed an activating gene signature, with high gene expression levels of CD69, CD83, and CXCR4 [50], as described previously [52,53,54]. Additionally, HBcAg-specific B cells in the blood of chronic HBV patients were also shown to have slightly higher CD69 protein expression compared to global memory B cells [60]. In HCV, both global CD27-positive and HCV E2-specific memory B cells have been found to highly express the liver-homing chemokine receptor CXCR3 compared to healthy controls [14,54,55,61].

For HBV, one study reported a significant increase in CXCR3 protein expression by B cells in the blood of chronic HBV patients compared to healthy controls [54], and transcriptomic analysis comparing HBV-specific B cells showed that CXCR3 was highly expressed in HBcAg-specific B cells compared to HBsAg-specific B cells [62]. It has been suggested that the increased expression of CXCR3 by B cells may be associated with the recruitment of these cells from peripheral blood to inflammatory sites of the liver where its ligand, IFN-γ-inducible protein-10 (IP-10), is produced [14,61]. However, the exact role of CXCR3 and CXCR4 in B cell activation or trafficking to the liver remains unknown. Despite the fact that studies show an activating B cell signature in chronic viral hepatitis infection, antibody responses to HBsAg remain impaired in chronic HBV infection [60,63], and glycoprotein E2-specific neutralizing antibody responses remain delayed in the acute phase of HCV infection [21,64]. It is important to note that, before the era of effective antiretroviral therapy, B cell hyperactivation and concomitant poor antibody responses were also widely reported in patients with chronic human immunodeficiency virus (HIV) infections [65,66].

## 6. Evidence of Intrahepatic B Cell Infiltration in Chronic Viral Hepatitis Infections

HBV and HCV induce distinct immune responses, but both show extensive infiltration with intrahepatic structures reminiscent of tertiary follicles. An immunohistochemical examination of liver biopsies showed that B cells can infiltrate the liver and accumulate during active clinical phases of chronic HBV [45,46,47]. B cells were abundant in the portal tract areas, and the highest density of cellular infiltrates and follicle-like structures or aggregates was observed in the liver biopsies of patients in the HBeAg-positive chronic hepatitis phase compared to other phases [45,46]. The follicle-like structures observed are less defined in chronic HBV compared to the more clearly defined intrahepatic lymphoid follicles typically observed in patients with chronic HCV infections [67,68]. So far, it remains unknown whether these structures play a role in supporting an effective immune response against HBV or HCV. However, recent studies on HBV have shown that Toll-like receptor 7 agonists can induce intrahepatic lymphoid aggregates of T and B cells in vivo and promote HBsAg-specific B cell responses in vitro [69,70]. In HCV, evidence of intrahepatic B cell proliferation, maturation in germinal centers, and co-localization of CD4 T follicular helper cells in portal tracts suggests a functional B-T cell environment in liver follicles during HCV infection [67,71,72]. Gaining more insight into the role of B cells in these structures may guide future strategies to improve therapies aimed at inducing an effective immune response against HBV or HCV.

## 7. Characterization of Virus-Specific B Cells in Chronic Viral Hepatitis Infections

Recent technological advances have enabled the isolation and characterization of antigen-specific B cells using antigen baits [53,60,63,73,74]. For HBV, a limited number of studies have characterized HBV-specific B cells by conventional flow cytometry through the gating of memory B cells and the subsequent labeling of antigen-specific B cells with fluorescently labeled HBsAg or HBcAg baits [53,60,63]. To avoid non-specific staining of these rare HBV-specific B cells as much as possible, double labeling was performed using two different fluorescently labeled baits, which ensured a higher specificity [53,60]. In general, HBcAg-specific B cells represent about 0.1–1.0% of total memory B cells in patients with chronic HBV infections, with <0.05% positivity observed in healthy controls [60]. HBsAg-specific B cell frequencies were typically around or lower than 0.1% of total memory B cells, ranging from 0.05 to 0.12% in chronic HBV patients while largely undetectable or <0.02% positivity in non-vaccinated healthy controls [53,60,63]. In the case of HCV, both envelope glycoproteins E1 and E2 are the primary target of neutralizing antibodies; the identification of HCV-specific B cells mainly focused on the detection of HCV E2-specific B cells [73,74]. Studies have, to a lesser extent, characterized HCV-specific memory B cells using E2 glycoprotein tetramers, reporting frequencies of <0.05% E2-positive B cells in healthy individuals and generally between 0.1 and 1.2% of E2-specific memory B cells in both HCV-infected subjects who had previously cleared infection or had established chronic infection [73,74]. Interestingly, one study showed that E2-specific memory B cells were only detected in three out of seven HCV-infected patients during the late acute phase of infection; however, upon longitudinal examination, E2-specific memory B cells were detected in all seven patients who subsequently developed a persistent HCV infection [73]. In addition, E2-specific antibody responses showed the same kinetics [73]. These results suggest a delayed HCV-specific B cell and humoral response during acute HCV infection, consistent with previous reports [21]. It has been suggested that, at least in some patients, the development or expansion of HCV E2-specific memory B cells is hindered during the acute phase of infection, which may be a factor contributing to the establishment of a chronic HCV infection [21,73]. Similar to HCV, HBsAg-specific B cells are also detectable in the blood of patients during an acute HBV infection, at low frequencies, and showing a tendency towards a decrease in relative frequency upon acute-resolving infection [53,63]. Moreover, our group showed that antigen-specific B cell responses in patients with chronic HBV infections are, for yet unknown reasons, predominately directed against HBcAg and not HBsAg [60]. A relative increase in the frequency and functional responses of HBcAg-specific memory B cells was associated with active clinical HBV phases characterized by elevated ALT levels [60]. Overall, weaker HBsAg-specific B cell responses and lower frequencies were observed in all four HBsAg-positive clinical phases of chronic HBV infection [60].

## 8. Enrichment of Atypical Memory B Cells in Chronic Viral Hepatitis Infections

It remains unknown why the humoral immune responses in chronic HBV patients are predominately directed against HBcAg and not HBsAg. The HBcAg is located within the nucleus or cytoplasm of infected hepatocytes, and is known to be highly immunogenic [75]. The enhanced immunogenicity can be explained by both its structure and its capability of eliciting both a T-cell-dependent as well as a T-cell-independent response [75,76]. On the contrary, the HBsAg antigen is actively secreted in blood by infected hepatocytes, and continuous exposure to high levels of HBsAg is postulated to be immunomodulatory, thereby contributing to a weak immune response against HBV in chronically infected patients [77,78,79,80]. B cell dysfunctions and the enrichment of so-called atypical memory (AtM) B cells are considered the key features of chronic viral hepatitis infections (Figure 3). It has been suggested that these may, at least in part, explain the defective anti-HBs antibody response observed in chronic HBV patients [53,63,81,82] and the delayed HCV E2-specific antibody response reported in the acute phase of HCV infection [64]. Since their discovery, AtM B cells have been reported at high frequencies in conditions of chronic antigen stimulation, such as autoimmune disease or infection with HIV and malaria [83,84,85]. Due to their initial discovery in chronic infections and autoimmune diseases, and their typical phenotype expressing high levels of inhibitory receptors, AtM B cells have been considered to be a population of exhausted B cells that arise due to chronic antigenic stimulation [86]. For both HBV and HCV, studies have reported an overall increased frequency of 4–17% AtM B cells within the total B cells in chronic infected patients, compared to 2–8% AtM B cells generally reported in heathy controls [53,63,82,87,88,89,90]. In both chronic HBV and HCV infections, AtM B cells are characterized by a low expression of CD21 and CD27 and high expression of T-Bet, CXCR3, CD11c, and FCRL5, similar to the previously described anergic CD21^low^ memory B cell subset in patients with HIV infection, with reported frequencies ranging from 14-56% of total B cells [84,88]. AtM B cells in chronic HBV patients have been shown to have impaired B cell receptor signaling, antibody production, and reduced IL-6 and TNF cytokine production compared to classical CD27-positive memory B cells, suggesting that AtM B cells may have a weaker antiviral function [63]. In chronic HCV infections, AtM B cells appear not to be exhausted in terms of antibody secretion but do appear to have exhausted proliferation [91].

Furthermore, it has been shown that HBsAg-specific B cells have defective antibody production and that, generally, 8% up to 60–80% of cells exhibit an AtM phenotype in chronic HBV patients with a higher expression of T-Bet and the inhibitory receptors FCRL5 and PD-1 compared to HBsAg-specific B cells in healthy vaccinated controls [62,63,82]. It is important to note that only 2–20% of HBsAg-specific B cells were enriched for the AtM phenotype in healthy vaccinated controls [62]. In addition, transcriptomic analysis comparing HBsAg- and HBcAg-specific B cells showed differences in gene expression profiles, showing the higher expression of *IL6R*, *CXCR3*, and *CD74* in HBcAg-specific B cells and *CD24*, *PECAM1*, *NFATC1*, and *TNFRSF13B* in HBsAg-specific B cells [62]. Although HBV-specific B cells differ in phenotype, the transcriptome profiles of HBsAg- and HBcAg-specific B cells were largely overlapping when compared to global memory B cells, both showing the high expression of AtM B-cell-associated genes (e.g., *FCRL3*, *FCRL5*, *SIGLEC6*, *CD84*, and *FCGR2B*) and genes linked to innate immune activation such as *MYD88*, *IFNA1/13*, *IFNA2*, and *IFNB1* [62]. For HCV, only a few studies so far have managed to detect and characterize HCV E2-specific B cells [61,64]. About 5–25% of E2-specific memory B cells in HCV infection have been shown to be enriched for the AtM phenotype by flow cytometry, and these cells, similar to HBsAg-specific B cells in chronic HBV, highly express FCRL5 and PD-1 compared to global memory B cells and healthy controls [61,64]. Additionally, the overexpression of FCRL5 and PD-1 on E2-specific memory B cells was associated with reduced anti-E2 plasma IgG levels when assessing a group of patients who were transitioning to a persistent HCV infection [64], suggesting exhausted antibody function in E2-specific B cells of chronic HCV patients. Despite the fact that a variable percentage of HBV-specific B cells (8–60%) and a small subset of HCV-specific B cells (5–25%) are considered exhausted [62,63,64,82], this does not fully explain the impaired anti-HBs antibody responses observed in all four HBsAg-positive clinical phases of chronic HBV infection [60], nor the delayed HCV E2-specific neutralizing antibody responses during the acute phase of HCV infection [21,64]. Although AtM B cells are generally thought to be exhausted, recent studies reported that AtM B cells do not always represent an exhausted B cell population [86,92,93,94,95,96]. Whether or not AtM B cells are part of a functional immune response may depend on the context, stage of infection, or the definitions used for B cell immune inhibition or exhaustion. Studies in HCV have, for example, reported that the expression of the exhaustion-associated marker FCRL4 in selected B cell subsets did not impair in vitro virus-induced B cell activation in patients with chronic HCV infections [57]. The enrichment of AtM B cells in chronic viral infections is accompanied by a simultaneous enrichment of hyperactivated B cells, as mentioned earlier. Therefore, we speculate that the continuous stimulation of B cells as a result of repeated antigen exposure could possibly explain both the enrichment of hyperactivated and AtM B cells during chronic infection. The exact role and function of AtM B cells in chronic viral hepatitis infections, and to what extent these are related to AtM B cells in other chronic viral infections, remain unknown.

## 9. Effects of Antiviral Treatment on B Cell Responses in Chronic Viral Hepatitis Infections

It is tempting to speculate that disbalances between functional, activated, and exhausted AtM B cell subsets are associated with treatment outcome, virological control, or (therapeutic) vaccination response, and that perhaps a shift in these balances might recover the antiviral B cell response. Some evidence points towards a possibility for the partial recovery of impaired anti-HBs antibody responses in chronic HBV patients by blocking the immune checkpoint inhibitor PD-1 on B cells [53,63,81], which can be further enhanced by suppressing HBsAg or inducing HBsAg seroconversion associated with a reversal of B cell hyperactivation and functional impairment [52,81]. Various siRNA-based therapeutic approaches aimed at suppressing HBsAg production from both integrated HBV DNA and cccDNA are currently being investigated in clinical trials [31]. However, the potential benefits of these approaches on B cell immune responses to HBV are not yet clearly understood. For HCV, the mechanisms responsible for progression to persistent infection are unclear, but unlike HBV, escape from adaptive immune responses can be achieved by the emergence of viral escape mutations that avoid recognition by antibodies and T cells [97]. Therefore, a broadly neutralizing antibody response may be important for HCV clearance [33,98]. It remains to be determined whether blocking immune checkpoint inhibitors could also be beneficial for chronic HCV patients to reinvigorate neutralizing antibody responses against HCV. Moreover, in HCV, it has been shown that sustained virological response to interferon-alpha therapy is associated with a reversal of the B-cell-activating signature in blood, mainly characterized by a decrease in CD69 and CD86 protein expression [59]. In contrast, the resolution of HCV infection with DAAs is associated with a marked reduction in the frequency of AtM and T-Bet-expressing B cells in blood [87,99], further suggesting that the type of treatment and subsequent outcome may be associated with a recovery of disbalances between functional, activated, and AtM B cell subsets in the blood of patients with chronic HCV infections. More specifically, for chronic HBV infections, it is known that HBcAg-specific B cell responses are significantly reduced during antiviral NUC treatment [60]. However, for HCV, it remains unknown how DAAs could affect HCV-specific B cell frequencies and functional responses in patients with chronic HCV infections.

## 10. Future Perspectives

Previous attempts using conventional methods such as flow cytometry for the characterization of HBV- and HCV-specific B cells have been challenging and have not yet provided us with a complete understanding of their role in chronic viral hepatitis infections. Technological advances in the field of next-generation sequencing now enable us to perform single-cell RNA sequencing on virus-specific B cells. This methodology will allow us to compare specific B cell populations in patients across different clinical phases of chronic HBV infection and stages of HCV infection before or after the initiation of antiviral treatment. This could provide us with detailed information on their functionality, and whether exhaustion phenomena are determining antigen-specific B cell responses in chronic viral hepatitis infection. Furthermore, by exploring the immune cell landscape in blood and liver at single-cell resolution, we may gain further insights into the role of B cells with respect to their anatomical location and their potential interaction with other immune cell subsets [100]. The collection of liver fine-needle aspirates poses minimal risk and discomfort to patients and enables the characterization of several B cell subsets in the liver [100,101,102]. The ability to electively and longitudinally sample liver fine-needle aspirates from patients without medical indication and intensively profile the immune landscape of the liver will enable us to identify biomarkers for intrahepatic immune activity in chronic viral hepatitis infections [100,103,104]. However, the relatively low frequency of B cells in the liver and even lower frequencies of virus-specific B cells in the blood of patients is considered one of the main limitations for future studies. Unravelling the role of B cells may guide strategies to improve therapies aimed at inducing an effective and long-lasting immune response against HBV and HCV in chronically infected patients. Furthermore, a better understanding of B cell biology in chronic viral hepatitis infections could have significant implications for the development of new therapies and vaccines to combat other chronic viral infections.
